# Association of perceived stress and coping strategies with depressive symptoms in students at a private medical college in islamabad

**DOI:** 10.1192/j.eurpsy.2021.872

**Published:** 2021-08-13

**Authors:** U. Zubair, S. Mansoor, T. Mansoor

**Affiliations:** 1 Oak, phoenix care center, Dublin, Ireland; 2 Psychiatry, FUMC, Islamabad, Pakistan

**Keywords:** coping strategies, depression, perceived stress.

## Abstract

**Introduction:**

The environment at medical colleges is competitive and typically generates higher stress levels. Both academic and psychosocial stresses appear to play a role, and the resourceful students who are able to employ effective coping strategies to deal with their stress are shown to outperform their peers in the academic settings.

**Objectives:**

Objective: To determine the Association of Perceived Stress and Coping Strategies with Depressive symptoms in students at a private medical college in Islamabad

**Methods:**

Fourth and Final year medical students of Foundation university medical college were enrolled in the study. Beck’s Depression Inventory was used to assess the depressive symptoms, Perceived Stress Scale (PSS) was the tool used to look for the perceived stress and the coping strategies were assessed using the Brief COPE Inventory. Association of Perceived Stress and Coping Strategies with Depressive symptoms and other sociodemographic factors was established.

**Results:**

Out of 262 medical students studied, 211 (80.5%) had no or mild depressive symptoms while 51 (19.5%) had moderate to severe depressive symptoms. 66 (25.2%) had low stress, 127 (48.4%) had moderate stress while 69 (26.3%) had high stress. Chi-square test revealed that perceived stress, self-distraction, active coping, denial, substance use, behavioral disengagement, positive reframing, acceptance, religion/ spirituality and self-blaming had statistically significant relationship with presence of depressive symptoms among the target population.

**Conclusions:**

Considerable number of medical students had presence of moderate to severe depressive symptoms in our study. Perceived stress and some specific kinds of coping strategies had significant association with presence of depressive symptoms among target population

